# Modeling and Preliminary Analysis of the Impact of Meteorological Conditions on the COVID-19 Epidemic

**DOI:** 10.3390/ijerph19106125

**Published:** 2022-05-18

**Authors:** Chenglong Sun, Liya Chao, Haiyan Li, Zengyun Hu, Hehui Zheng, Qingxiang Li

**Affiliations:** 1 School of Atmospheric Sciences and Key Laboratory of Tropical Atmosphere-Ocean System, Ministry of Education, Sun Yat-Sen University, Zhuhai 519082, China; sunchlong@mail2.sysu.edu.cn (C.S.); chaoly@mail2.sysu.edu.cn (L.C.); lihaiyan@cau.edu.cn (H.L.); 2 Xinjiang Institute of Ecology and Geography, Chinese Academy of Sciences, Urumqi 830011, China; huzengyun@ms.xjb.ac.cn; 3 National Institute of Environmental Health, Chinese Center for Disease Control and Prevention, Beijing 100021, China

**Keywords:** COVID-19 epidemic, meteorological drivers, modeling, LgR model, PLSR model

## Abstract

Since the COVID-19 epidemic outbreak at the end of 2019, many studies regarding the impact of meteorological factors on the attack have been carried out, and inconsistent conclusions have been reached, indicating the issue’s complexity. To more accurately identify the effects and patterns of meteorological factors on the epidemic, we used a combination of logistic regression (LgR) and partial least squares regression (PLSR) modeling to investigate the possible effects of common meteorological factors, including air temperature, relative humidity, wind speed, and surface pressure, on the transmission of the COVID-19 epidemic. Our analysis shows that: (1) Different countries and regions show spatial heterogeneity in the number of diagnosed patients of the epidemic, but this can be roughly classified into three types: “continuous growth”, “staged shock”, and “finished”; (2) Air temperature is the most significant meteorological factor influencing the transmission of the COVID-19 epidemic. Except for a few areas, regional air temperature changes and the transmission of the epidemic show a significant positive correlation, i.e., an increase in air temperature is conducive to the spread of the epidemic; (3) In different countries and regions studied, wind speed, relative humidity, and surface pressure show inconsistent correlation (and significance) with the number of diagnosed cases but show some regularity.

## 1. Introduction

The emergence of the COVID-19 virus since the end of 2019 has caused a global pandemic of novel coronavirus outbreaks [1]. Novel coronavirus causes fever in humans, induces pneumonia, and then leads to respiratory failure in patients [2]. Studies have shown that novel coronavirus is transmitted mainly by droplets containing the virus or by contact with infected surfaces [3]. Meteorological factors such as air temperature and relative humidity may influence the spread of the novel coronavirus outbreak by affecting the survival of the virus during transmission [4]. It has been suggested that climatic factors are effective predictors of coronavirus disease [5].

Many studies have been conducted on the correlation between meteorological elements and the COVID-19 outbreak [6,7,8,9,10,11,12,13,14,15,16,17,18,19,20,21,22,23,24,25,26]. Several studies have shown [6,7,8] that temperature is negatively correlated with the number of new cases and deaths per day, with a corresponding decrease in the number of new cases and deaths per day for every 1 °C increase in air temperature. Some previous laboratory studies [9,10,11,12,13] also found that the survival time of the virus became correspondingly shorter with increasing temperature. However, other studies [14,15,16] showed a positive correlation between the number of COVID-19 infections and temperature, while others found no significant correlation between novel crown pneumonia and temperature [17,18,19]. Alternatively, it was found that there may be a relatively suitable temperature range for the novel crown pneumonia [20,21,22]. Similarly, relative humidity may be one of the essential meteorological factors affecting the transmission of COVID-19. Related studies [23] showed that absolute humidity was positively correlated with the exponential increase in the development of the COVID-19 epidemic and that the survival and transmission of the COVID-19 virus were more favorable in humid environments. In contrast, other studies [24,25] concluded that humidity was negatively correlated with novel crown pneumonia. In general, wind speed has an important influence on the transport dispersion of atmospheric pollutants, which may affect the spread of COVID-19 virus particles by diluting their concentration and changing their trajectory. However, several studies have shown that the correlation between wind speed and COVID-19 transmission is not statistically significant; some individual studies found a non-linear relationship between wind speed and COVID-19 transmission [26]. Therefore, studies on the influence of meteorological factors on the spread of COVID-19 outbreaks are inconsistent and subject to significant uncertainty. On the one hand, many studies have used different methods and areas of interest; on the other hand, most importantly, the factors that may influence the transmission of the COVID-19 epidemic are complex. Most existing studies have failed to truly separate the part of the epidemic development process driven by meteorological factors.

Different mathematical models are often used to explore the developmental process of infectious diseases [27,28,29,30]. Several studies have used the SIR model and its extensions to explore novel crown pneumonia outbreaks’ basic patterns and forecasts in different countries and regions. However, these models require more parameters to be set. They are still sensitive to the choice of each parameter, making it difficult to understand the dynamics in the absence of detailed observational data. Logistic regression (LgR) models with relatively little complexity and sensitivity have been used to explore the transmission of epidemics in different countries and regions, including recent novel crown epidemics [31,32,33,34]. To accurately investigate the influence of meteorological factors on the COVID-19 epidemic, this paper considers the characteristics of the LgR model. It proposes a combinatorial modeling approach that is less dependent on parameter settings and separates the influence of meteorological factors on the transmission of the epidemic as accurately as possible while considering the exclusion of the development pattern of the epidemic itself. By using this method, the possible influence of each of the meteorological factors such as air temperature, relative humidity, wind speed, and surface pressure on the transmission of the COVID-19 epidemic was systematically analyzed.

The sections of this paper are organized as follows: Section 2 introduces the research data and modeling methods. Section 3 presents the modeling analysis of the epidemic transmission model itself and the modeling analysis of the influence of meteorological factors on the epidemic transmission. Section 4 provides some discussions on the rationality of some specific results. Section 5 is the conclusions and outlook of this paper.

## 2. Materials and Methods

### 2.1. Research Data

#### 2.1.1. COVID-19 Epidemic Data

The data collected in this paper on COVID-19 in selected countries and regions of the world are from Johns Hopkins University. More detailed regional (provinces and cities in China, states in the United States) epidemic data are available for two countries (China, United States) from China National/Provincial health councils and from the website: https://www.kaggle.com/fireballbyedimyrnmom/us-counties-covid-19-dataset (accessed on 1 December 2021). The epidemic variables collected include the cumulative number of infections and the number of new diseases for each country (region) on a day-by-day basis. The date of the first local outbreak is used as the starting date of the study, with data collected up to 30 September 2021.

#### 2.1.2. Meteorological Data

The meteorological factors data used in this paper are the European Centre for Medium-Range Weather Forecasts (ECMWF) ERA5 reanalysis data [35]. ERA5 reanalysis data were generated using four-dimensional data assimilation technology with a resolution of 0.25° × 0.25° in latitude and longitude. ERA5 reanalysis data can be downloaded at: https://cds.climate.copernicus.eu/cdsapp#!/search?type=dataset (accessed on 1 December 2021). Comparative analysis shows that the ERA5 reanalysis data has a good reproducibility for global and regional climate change [36]. The meteorological elements in the ERA5 reanalysis data used in this paper include: 2 m air temperature, 2 m dew point temperature, 10 m longitudinal and latitudinal wind, and surface pressure. All of which are reorganized into daily average values. ERA5 does not provide relative humidity data. Therefore, the relevant elements are calculated based on the Equations (1) and (2).

According to the Goff-Grattch correction formula [37]:

(1) $$E=E_{0}\times 10^{\frac{aT}{b+T}}$$

Calculate relative humidity:

(2) RH=(E/Es)×100

where *E*_0_ is 6.10695, *a* is 7.59271, *b* is 240.72709, and *E* and *Es* are the actual and saturated water vapor pressure, respectively.

### 2.2. Modeling Method

This paper investigates the effect of meteorological factors on the COVID-19 epidemic using a combined model of the LgR model and the partial least squares regression (PLSR) model. According to the shape of the LgR model, it represents a single typical epidemic cycle [31,38]. The difference between the actual epidemic and the logistic simulation of the epidemic (i.e., the residuals of the regression equation) is considered as the contribution due to factors other than the transmission laws of the epidemic itself. The PLSR model is then adopted to separate the meteorological drivers in the residuals and to determine the effect of each meteorological factor studied on COVID-19.

#### 2.2.1. COVID-19 Self-Transmission Model

The growth pattern of the number of infectious diseases in nature is similar to an “S-shaped” curve [37], with roughly exponential growth in the initial stage, followed by saturation and slowing down as the number increases, and finally reaching maturity when growth basically stops.

The expression of the LgR model function is:

(3) $$P(t)=\frac{KP_{0}e^{rt}}{K+(P_{0}e^{rt}-1)}$$

where *P*_0_ is the initial population value, which represents the initial number of infections in the infectious disease model; *K* is the environmental capacity, which means the maximum cumulative number of infections in the model; *r* is a measure of how fast the curve changes, which represents the disease transmission rate in the infectious disease model; *t* is time, and *P(t)* is the population size over time, which means the cumulative number of infections over time in the infectious disease model. The value of *r* measures how fast the curve changes in the traditional LgR model. If the value of *r* is large, the epidemic is developing rapidly; conversely, the slower the epidemic is growing.

The goodness of fit is used to express the behavior of the LgR model (how the fitted value fits the observations). The maximum value of R² is 1. The closer the value of R² is to 1, the better the fit to the observations; conversely, the smaller the value of R² is, the worse the fit to the observations.

Let y be the value to be fitted, its mean value is $\bar{y}$, and the fitted value is $\hat{y}$.

Sum of squares total (*SST*):

(4) $$SST=\sum_{i=1}^{n}{\left(y_{i}-\bar{y}\right)}^{2}$$

Sum of squared regression (*SSR*):

(5) $$SSR={\sum_{i=1}^{n}\left(\hat{y_{i}}-\bar{y}\right)}^{2}$$

Sum of squared errors (*SSE*):

(6) $$SSE={\sum_{i=1}^{n}\left(y_{i}-\hat{y_{i}}\right)}^{2}$$

The goodness of fit is:

(7) $$R^{2}=1-\frac{SSE}{SST}$$

#### 2.2.2. Modeling the Influence of Meteorological Factors on COVID-19

To avoid the application limitations of ordinary multiple linear regression (MLR), S. Wold and C. Albano et al. first proposed the partial least squares regression (PLSR) method in 1983 [39]. When there are multiple correlations between variables, using ordinary least squares for MLR analysis can jeopardize parameter estimates, amplify model errors, and undermine model robustness. PLSR analysis extracts the variables with the strongest explanatory power for the dependent variable by decomposing and filtering the data information, thus effectively overcoming the undesirable effects of multiple correlations of variables in system modeling. The PLSR model performs well in the separation of the influence of the external forcings on surface air temperature and precipitation [40,41,42]. Unlike the traditional MLR analysis method, it first obtains the standardized matrices of the independent and dependent variables through standardization, then performs principal component analysis on the independent variables, extracts the principal component corresponding to the largest eigenvalue that is most closely related to the dependent variable and the corresponding load vector, and uses this principal component to regress with the dependent variable to find the respective residual matrices; then performs similar processing on the residual matrices, and so on analogously.

The explained variance of the predicted dependent variable calculated from the residuals is used to measure the stability of the equation estimated by the partial regression. The number of selected principal components is determined in this way [43]. The model expression is:

(8) $$E_{0}=\sum_{i=1}^{s}t_{i}p_{i}^{\prime }$$

(9) $$F_{0}=\sum_{i=1}^{s}t_{i}r_{i}^{\prime }+F_{s}$$

where $E_{0}$ denotes the normalization matrix of the independent variable, $E_{0}={\left(E_{01},\cdots ,E_{0p}\right)}_{n\times p}$, $F_{0}$ denotes the normalization matrix of the dependent variable, $F_{0}={\left(F_{01},\cdots ,F_{0p}\right)}_{n\times q}$, s denotes the number of components extracted from the original variables, $t_{i}$ denotes the principal component vector of the extracted independent variable matrix one at a time, $p_{i}$ denotes the load vector of the dependent variable, $r_{i}$ denotes the projection vector of the dependent variable on the principal component axis, $F_{s}$ denotes the residual matrix, and the symbol (′) denotes the transpose.

Since $t_{1}\cdots t_{s}$ can all be expressed as a linear combination of $E_{01}\cdots E_{0p}$, Equations (8) and (9) can be reduced to the form of the regression equation of the standardized dependent variable $y_{k}^{*}=F_{0k}$ on the standardized independent variable $x_{j}^{*}=E_{0j}$. The expression is:

(10) $$y_{k}^{*}=\sum_{j=1}^{p}\alpha_{kj}x_{j}^{*}+F_{sk}$$

where k=1,⋯,q, j=1,⋯,p, $\alpha_{kj}$ denotes the standardized coefficient of the *j*th independent variable with respect to the *k*th dependent variable, $F_{sk}$ denotes the kth column of the residual matrix, and (*) denotes the standardization treatment.

The variable importance of projection (*VIP*), is the explanatory power of the independent variable $x_{j}$ on the set of dependent variables Y. It is defined as:

(11) $$VIP_{j}=\sqrt{\frac{p}{\sum_{i=1}^{s}R\left(Y,t_{i}\right)}\sum_{i=1}^{s}R\left(Y,t_{i}\right)w_{ij}^{2}}$$

where p denotes the number of independent variables, $R\left(Y,t_{i}\right)$ denotes the explanatory power of $t_{i}$ on the set of dependent variables Y as the square of their correlation coefficients, $w_{ij}$ denotes the jth component of the axis $w_{i}$, and *w* denotes the eigenvector of the largest eigenvalue of the matrix $E_{i-1}^{\prime }F_{i-1}F_{i-1}^{\prime }E_{i-1}$.

The *VIP* value can be used to filter the variables that contribute more to the model. A *VIP* value greater than 1 indicates that the independent variable has a more critical explanatory role for the dependent variable; a *VIP* value of 0.5–1 suggests that the importance of the explanatory role is not entirely clear and requires additional samples or judgment based on other conditions; a *VIP* value less than 0.5 indicates that the explanation of the independent variable for the dependent variable is meaningless [44,45].

## 3. Results

### 3.1. Characteristics of COVID-19 Transmission in Selected Countries and Regions

**Figure 1:**
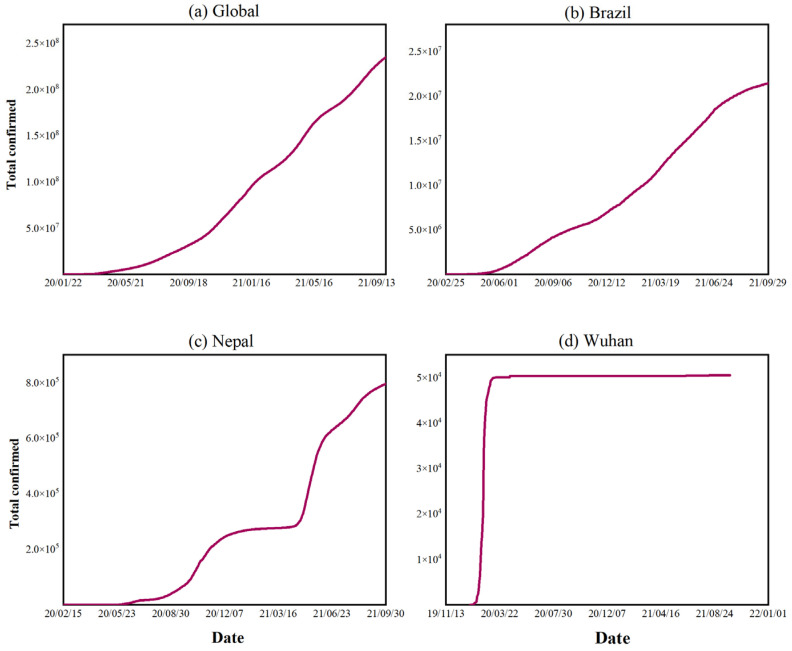
Global and typical national and regional epidemic trends. Figures (**a**–**d**) represent the cumulative confirmed cases of the COVID-19 globally, in Brazil, Nepal, and Wuhan, respectively.

Since the outbreak of COVID-19, there has been a significant impact on the economy and public health system. The attack is still in the process of developing in many countries and regions. Three main types characterize the development of the COVID-19 epidemic in selected countries and regions worldwide. The first type is the “continuous growth” type, Figure 1a shows the development trend of the global cumulative confirmed cases, and by 30 September 2021, the cumulative confirmed cases globally of the COVID-19 epidemic exceed 200 million, showing a continuous rapid growth trend. Figure 1b shows the development trend of the COVID-19 epidemic in Brazil, which exhibits similar development characteristics to the global. The second type is the “staged shock”, as shown in Figure 1c. The development of the epidemic in Nepal shows a multi-stage character, with slow growth or even stagnation after a certain period, followed by rapid growth again. The third type is the “finished”, as shown in Figure 1d, which indicates the development trend of confirmed cases in Wuhan since the outbreak of COVID-19. In the early stage of development, the COVID-19 epidemic spread rapidly. The epidemic was effectively controlled in a short period due to the strong control measures taken by the government, so the region experienced a rapid “finished” process.

**Figure 2:**
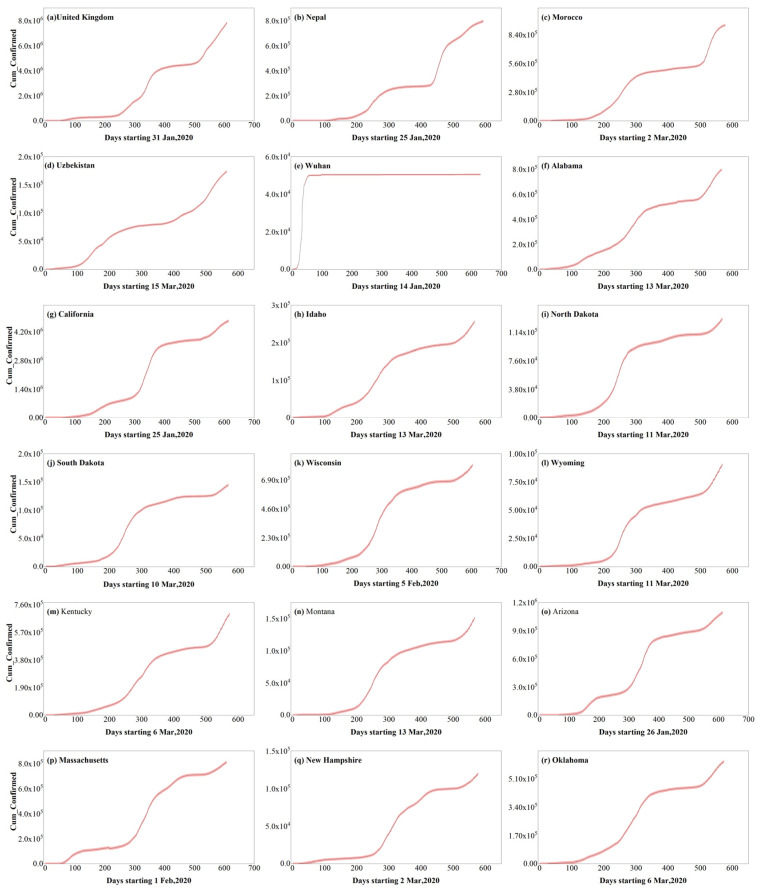
Cumulative confirmed cases in the countries and regions studied. Figure (**a**–**r**) represents the cumulative confirmed cases of COVID-19 in our study area and time respectively.

To better analyze the characteristics of the spread of the epidemic, accurately simulate the transmission pattern of the COVID-19 epidemic within a certain period, and explore the influence of meteorological factors on the spread of the COVID-19 epidemic, this paper selects relevant countries and regions (involving Asia, Europe, Africa, and North America) with a large spatial span, a certain degree of representativeness, and whose epidemic spread has a certain periodicity (the second and third types mentioned above) for the study. Table 1 presents the “epidemic cycle” time window for developing the epidemic in the countries and regions selected for this paper. We chose the first day of recording as the start date of the study, while the termination date was chosen based on the criterion that the cumulative number of diagnoses reached a relatively flat stage of development. We also selected different termination dates for sensitivity testing during the relatively flat phase (Supplementary Information Table S2). Among these countries (regions or cities), Wuhan has the finished type, while the other countries and regions have the staged shock type (Figure 2). Nepal, Uzbekistan, and Wuhan are located in Asia, the United Kingdom in Europe, Morocco in Africa, and the U.S. states in North America.

### 3.2. Modeling of the COVID-19 Self-Development

**Figure 3:**
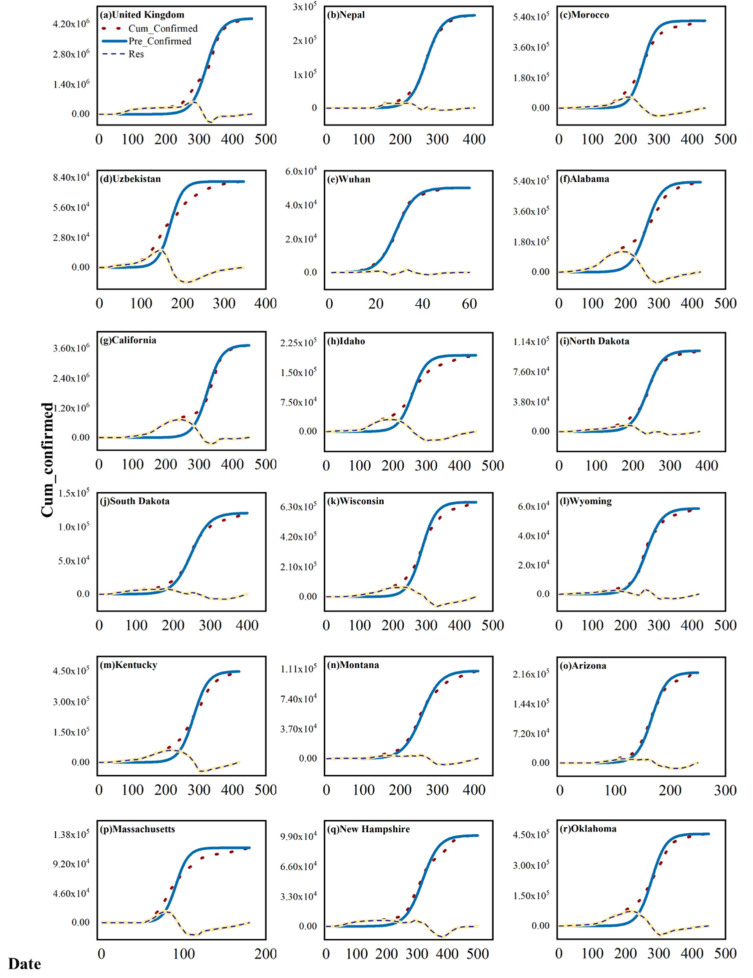
Figures (**a**–**r**) represents the fitting of cumulative confirmed cases of COVID-19 with the LgR model in different countries and regions.

Figure 3 represents the fit of the LgR model to the cumulative confirmed cases of the COVID-19 epidemic for different countries and regions studied during their “epidemic cycles” (Table 1). The dotted lines represent the observed cumulative confirmed cases of the epidemic. The blue curves represent the cumulative confirmed cases fitted according to the LgR model. The orange curves represent the residuals between the observed cumulative confirmed cases and the cumulative confirmed cases simulated by the LgR model. The parameter values and goodness of fit of the LgR model for the different countries and regions studied are given in Table 2. As shown in Table 2, the goodness of fit of the LgR models for all 18 countries and regions reached above 0.9, indicating that the model has a relatively good simulation of the transmission process of the studied COVID-19 epidemic and can better capture the transmission characteristics and trends of the epidemic.

### 3.3. Separation of the Influence of Meteorological Drivers on the Transmission of the COVID-19

**Figure 4:**
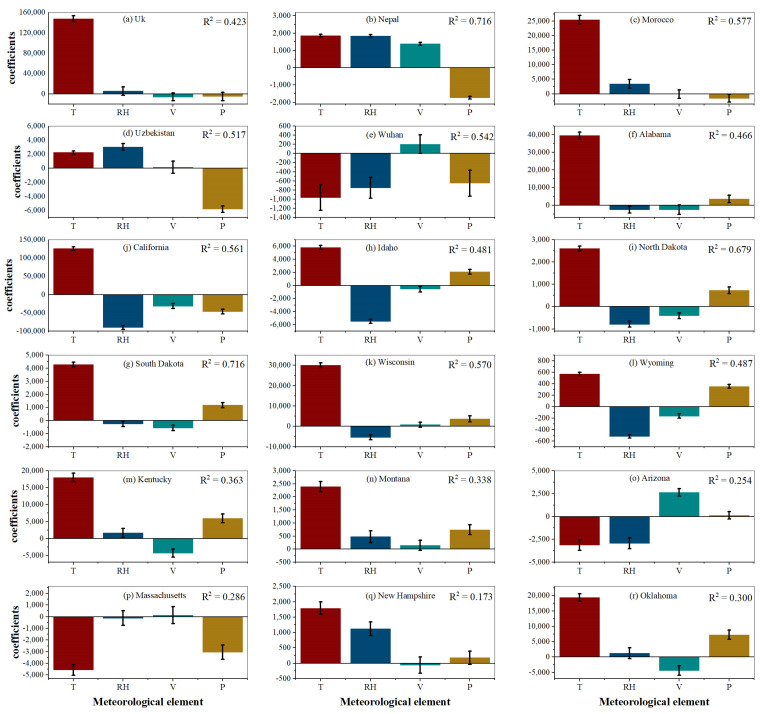
Figures (**a**–**r**) represents the standardized coefficients of the PLSR for different countries and regions and their significance intervals at 5% confidence level.

The PLSR modeling analysis was then performed to separate the effects of meteorological factors on the transmission of the COVID-19 epidemic. We use the residuals of the LgR modeling relative to the actual spread of the epidemic in Figure 3 above as the dependent variables and the corresponding air temperature, relative humidity, wind speed, and surface pressure over the same period as independent variables. The standardized coefficients of the PLSR model and their significance intervals at 5% confidence are given in Figure 4. The magnitude of the coefficients indicates the relative importance of this meteorological factor on the variation of the residuals.

As shown in Figure 4, the PLSR model can well capture the meteorological information in most study areas and explain the influence of meteorological factors on the spread of the COVID-19 epidemic to a certain extent, with 11 countries and regions explaining variance above 0.45. Figure 4 also shows the standardized coefficients and their significance at a 5% level for each meteorological factor obtained from PLSR modeling with air temperature, relative humidity, wind speed, and surface pressure as the driving factors for each country and region. The effect of air temperature on the COVID-19 epidemic is significant in all the countries and regions studied. The coefficients of PLSR of air temperature in Wuhan, Arizona, and Massachusetts were negative, indicating a negative correlation between temperature and the COVID-19 epidemic in these three regions, i.e., an increase in temperature is detrimental to the transmission of the COVID-19 epidemic. The coefficients of PLSR of air temperature in the remaining countries and regions studied were positive, indicating that the increase in air temperature is favorable to the transmission of the COVID-19 epidemic. We investigated the influence of air temperature on the development of the epidemic at different lag days, including 5-day, 7-day, and 14-day lag conditions. The results show that the effect of air temperature on the epidemic under different lag time conditions is consistent with the results of our chosen reference date, showing great stability. We also chose different periods of time to explore the stability of the effect, and the results show that the air temperature factor has a consistent effect on the epidemic (Supplementary Information Tables S1 and S2).

Relative humidity significantly affected the COVID-19 epidemic in the regions studied, except for the UK, Massachusetts, South Dakota, Kentucky, and Oklahoma states. For Nepal, Morocco, Uzbekistan, Montana, and New Hampshire, the coefficient of PLSR relative humidity was positive, indicating that relative humidity showed a positive relationship with the COVID-19 epidemic. For the other regions, the coefficient of PLSR relative humidity was negative, indicating that relative humidity was negatively correlated with the COVID-19 transmission. Wind speed was significantly correlated with the COVID-19 spread in Nepal, California, North Dakota, South Dakota, Wyoming, Kentucky, Arizona, and Oklahoma. For other areas, wind speed did not significantly correlate with the COVID-19 spread. For surface pressure, the UK, Arizona, Morocco, Alabama, and New Hampshire were not significantly correlated with the COVID-19 epidemic, and all other countries and regions studied showed significant correlations between surface pressure and the COVID-19 epidemic. Nepal, Uzbekistan, Wuhan, California, and Massachusetts showed significant negative correlations with COVID-19, while the other regions showed significant positive correlations. Similarly, we explore the effects of the relative humidity, wind speed, and surface pressure on the development of the epidemic under different lag times, including 5-day, 7-day, and 14-day lag conditions. The results show that relative humidity, wind speed, and surface pressure show some differences in the significance and correlation of meteorological factors in some countries and regions at different lag days compared to the study dates, but most of them have consistent effects at different lag days. Among them, the surface pressure factor shows the strongest consistency and differences only in Morocco and California. Similarly, the performance of these three meteorological factors at different times does not show the same exact conclusions as the temperature, but there is still consistency in some countries and regions (Supplementary information Tables S1 and S2).

## 4. Discussion

### 4.1. Possible Explanations for the Influence of Different Meteorological Factors on COVID-19

Air temperature is significantly correlated with the COVID-19 epidemic in the countries and regions studied (Figure 4), which is consistent with the findings of most previous studies [46,47,48,49]. Except for Wuhan, Arizona, and Massachusetts, where the air temperature factor negatively correlated with the spread of COVID-19, all of the other countries and regions showed a positive correlation between the air temperature and the COVID-19 diagnosed cases. The inconsistent correlation with the epidemic in the studied areas might be related to the occurrence and duration of the epidemic, the epidemic sample size may also affect the stability of the model [50]. There may be special reasons for the negative correlation between air temperature and epidemic in the three regions mentioned above: firstly, as the virus appears in winter, there is a process of adaptation to meteorological factors in the short term, and its infectiousness may be suppressed as the air temperature rises; Secondly, in a short time, it cannot be excluded that strict human control measures have had some impact on statistical relationships. For the other areas studied, the sample size was more extensive as the study became more prolonged. Therefore the results were more stable and representative, most likely reflecting the true impact of air temperature on the COVID-19 outbreak. One possible explanation for the positive correlation between air temperature and COVID-19 is that people are more likely to go out and be exposed to environments with the virus when air temperatures rise, thereby increasing the risk of infection [16].

The effect of humidity on virus transmission may be that the dry and humid conditions of the air affect the tiny droplets exhaled by the human body, affecting the attachment and replication of the virus and its transmission through evaporation and deposition. Relative humidity also can affect COVID-19 virus survival by affecting aerosols and thus COVID-19 virus survival [51]. The results of this paper found that the effect of relative humidity on COVID-19 outbreaks was significant in thirteen countries and regions, and most regions showed a negative correlation, similar to the results of some previous studies [24,25]. The lower the humidity and the drier the air, the smaller the aerosol condensation nuclei, and the smaller infectious aerosols will remain in the air longer. They may enhance the spread of the outbreak [52].

It was found that wind speed may affect airborne suspended particles of the virus and thus influence the development of the COVID-19 outbreak [53]. This paper found that wind speed did not affect the epidemic to the same extent in different regions. Its significance and correlations with the epidemic were not completely uniform. In some areas where the effect of wind speed was significant, the wind speed was negatively correlated with the epidemic. The possible explanation is that higher wind speeds have better air circulation and a more substantial dilution effect on airborne virus particles, thus attenuating the spread of the epidemic.

Surface pressure may affect the duration of virus suspension in the air by influencing the generation and extinction of weather systems and, in turn, the spread of the virus. Related studies have shown that the effect of surface pressure on the development of the epidemic can vary in different study areas [53]. This paper found that the effect of surface pressure on the COVID-19 epidemic is significant in thirteen countries and regions. Still, the correlations were not consistent across regions, which may also be influenced by other socioeconomic and other factors, and therefore need to be further studied.

In addition, since the emergence of the COVID-19 virus, the virus is still under constant mutation and has undergone α mutation, β mutation, γ mutation, omicron, etc. The possible differences in the effects of meteorological factors on different mutated viruses in different countries and regions may be one of the reasons for the incomplete consistency of the impact of meteorological conditions on the spread of the epidemic.

### 4.2. About the Fitting Effect of the PLSR Model

Based on the current study, the LgR model is able to fit well the transmission pattern of the epidemic itself (Table 2, R^2^ reached above 0.9). The explanation variances have been reduced when using the PLSR model to explore the effects of meteorological factors (air temperature, relative humidity, wind speed, and surface pressure) on the COVID-19 epidemic. Figure 4 shows that the PLSR models with meteorological factors as independent variables fit well (R^2^ > 0.45) for most countries and regions. While for a small number of countries and regions, the models fitted with a small R^2^ (R^2^ < 0.45). Possible explanations for this result are: (1) The residual series of the LgR model and the actual number of epidemics include, in addition to meteorological factors, factors such as socioeconomic conditions (economic level, medical level), interventions (individual health habits, regional health decisions), regional natural conditions, population movements (population differences, population characteristics and density), etc., which are not taken into account in the model; (2) The additional patients including the timely conversion of asymptomatic infected individuals to confirmed cases, the external input cases, etc. may also make the PLSR modeling results subject to a degree of uncertainty due to local differences in detection capacity.

Even so, at spatial scales (e.g., cities, regions, and countries of similar spatial extent in this paper), the PLSR model is still better able to summarize the correlation between meteorological factors and COVID-19 transmission over a complete epidemic cycle and separate the degree of influence of different meteorological factors on the COVID-19 epidemic.

## 5. Conclusions

The paper uses a combination of the LgR and PLSR models to investigate the meteorological drivers of the COVID-19 epidemic. The LgR model fits the developing laws of the epidemic itself, and the PLSR model is then used to explore the impact of each meteorological factor on the development of the epidemic. The following conclusions are drawn:

(1) Different countries and regions have different characteristics of transmission of the COVID-19, showing spatial inhomogeneity, but they can be roughly classified into three types (“continuous growth”, “staged shock”, and “finished”) according to the characteristics of development stages.
(2) Air temperature is the most significant factor associated with the transmission of the COVID-19 epidemic, with regional variations in air temperature and the transmission of the epidemic in most regions showing a significant positive correlation at a 95% confidence level.
(3) In the different countries and regions studied, there are significant correlations between the epidemic diagnosed cases and relative humidity/ wind speed and surface pressure in some areas. In general, increases in relative humidity/ wind speed and low surface pressure are not conducive to the transmission of the epidemic.

Due to the time and space limitations of the study, the conclusions obtained in this paper still have some limitations: We do not consider the influence of factors other than meteorological factors, which may also play a moderating role in developing the COVID-19 epidemic. For example, further studies will also need to consider incorporating potential risk factors such as socioeconomic conditions and human control in each country and region into the model to explore the more precise influence of meteorological factors on the COVID-19 epidemic.

## Figures and Tables

**Table 1:** Time windows for the different countries and regions studied.

Countries and Regions	Time Windows
United Kingdom	31 January 2020–2 May 2021
Nepal	25 January 2020–28 February 2021
Morocco	2 March 2020–14 May 2021
Uzbekistan	15 March 2020–24 February 2021
Wuhan	14 January 2020–13 March 2020
Alabama	13 March 2020–11 May 2021
California	25 January 2020–18 April 2021
Idaho	13 March 2020–4 June 2021
North Dakota	11 March 2020–25 March 2021
South Dakota	10 March 2020–13 April 2021
Wisconsin	5 February 2020–29 April 2021
Wyoming	11 March 2020–4 May 2021
Kentucky	6 March 2020–29 April 2021
Montana	13 March 2020–26 April 2021
Arizona	26 January 2020–1 October 2020
Massachusetts	1 February 2020–29 July 2020
New Hampshire	2 March 2020–14 July 2021
Oklahoma	6 March 2020–29 May 2021

**Table 2:** LgR model parameters and goodness of fit for different countries and regions.

Regions	K	P0	r	R^2^
United Kingdom	4,435,831	2	0.046	0.976
Nepal	274,143	1	0.047	0.996
Morocco	514,705	1	0.052	0.979
Uzbekistan	79,749	1	0.067	0.936
Wuhan	49,994	45	0.253	0.999
Alabama	531,404	6	0.044	0.906
California	3,718,367	1	0.046	0.938
Idaho	192,870	1	0.047	0.950
North Dakota	102,230	1	0.049	0.992
South Dakota	120,154	5	0.041	0.988
Wisconsin	659,812	1	0.047	0.977
Wyoming	58,367	1	0.042	0.994
Arizona	219,214	1	0.074	0.906
Kentucky	446,773	1	0.046	0.961
Montana	108,227	4	0.040	0.993
Massachusetts	116,684	1	0.130	0.958
New Hampshire	99,840	1	0.036	0.981
Oklahoma	452,777	1	0.047	0.965

## Supplementary Materials

The following supporting information can be downloaded at: https://www.mdpi.com/article/10.3390/ijerph19106125/s1, Table S1: Results At Different Lag Days; Table S2: Results For Different Time Duration.
