# Supplementary material for: Modeling and Preliminary Analysis of the Impact of Meteorological Conditions on the COVID-19 Epidemic
Source: Int J Environ Res Public Health. 2022 May 18;19(10):6125. doi: 10.3390/ijerph19106125 (PMC9140896; doi:10.3390/ijerph19106125)
Supplement: Supplementary file 1 [file ijerph-19-06125-s001.zip › ijerph-1717990-supplementary.pdf]

Table S1. Results At Different Lag Days.

**Regions**	Lag Days	R2(PLSR)	Significant factors and correlations
UK	0	0.423	T（+）
	5	0.437	T（+）
	7	0.440	T（+）
	14	0.457	T（+）、RH（-）
Morocco	0	0.577	T（+）、RH（+）
	5	0.620	T（+）、RH（+）、P（-）
	7	0.636	T（+）、P（-）
	14	0.678	T（+）、P（-）
Nepal	0	0.716	T（+）、RH（+）、V（+）、P（-）
	5	0.718	T（+）、RH（+）、V（+）、P（-）
	7	0.716	T（+）、RH（+）、V（+）、P（-）
	14	0.689	T（+）、RH（+）、V（+）、P（-）
Uzbekistan	0	0.517	T（+）、RH（+）、P（-）
	5	0.478	T（+）、RH（+）、P（-）
	7	0.452	T（+）、RH（+）、P（-）
	14	0.361	T（+）、RH（+）、P（-）
Wuhan	0	0.542	T（-）、RH（-）、P（-）
	5	0.369	T（-）、RH（+）、P（-）
	7	0.450	RH（+）、V（-）、P（+）
	14	0.264	T（-）、RH（-）、V（-）、P（-）
Alabama	0	0.466	T（+）、P（+）
	5	0.515	T（+）、RH（-）
	7	0.532	T（+）、RH（-）
	14	0.586	T（+）、RH（-）
Arizona	0	0.254	T（-）、RH（-）、V（+）
	5	0.278	T（-）、RH（-）、V（+）
	7	0.282	T（-）、RH（-）、V（+）
	14	0.314	T（-）、RH（-）
California	0	0.561	T（+）、RH（-）、V（-）、P（-）
	5	0.642	T（+）、RH（-）、V（-）
	7	0.668	T（+）、RH（-）、V（-）
	14	0.735	T（+）、P（-）
Idaho	0	0.481	T（+）、RH（-）、P（+）
	5	0.573	T（+）、RH（-）、V（+）、P（+）
	7	0.603	T（+）、RH（-）、P（+）
	14	0.685	T（+）、RH（-）、P（+）
Kentucky	0	0.363	T（+）、V（-）、P（+）
	5	0.430	T（+）、RH（+）、P（+）
	7	0.451	T（+）、RH（+）、P（+）
	14	0.517	T（+）、P（+）

Massachusetts	0	0.286	T（-）、P（-）
	5	0.232	T（-）、P（-）
	7	0.210	T（-）、P（-）
	14	0.138	T（-）、RH（+）
Montana	0	0.338	T（+）、RH（+）、P（+）
	5	0.361	T（+）、P（+）
	7	0.366	T（+）、P（+）
	14	0.395	T（+）、P（+）
New Hampshire	0	0.173	T（+）、RH（+）
	5	0.210	T（+）、RH（+）
	7	0.227	T（+）、RH（+）
	14	0.280	T（+）、RH（+）
North Dakota	0	0.679	T（+）、RH（-）、V（-）、P（+）
	5	0.723	T（+）、RH（-）、P（+）
	7	0.726	T（+）、RH（-）、P（+）
	14	0.718	T（+）、RH（-）、V（-）、P（+）
Oklahoma	0	0.300	T（+）、V（-）、P（+）
	5	0.366	T（+）、RH（+）、V（-）、P（+）
	7	0.391	T（+）、RH（+）、V（-）、P（+）
	14	0.467	T（+）、RH（+）、V（-）、P（+）
South Dakota	0	0.716	T（+）、V（-）、P（+）
	5	0.751	T（+）、P（+）
	7	0.758	T（+）、P（+）
	14	0.757	T（+）、P（+）
Wisconsin	0	0.570	T（+）、RH（-）、P（+）
	5	0.614	T（+）、V（+）、P（+）
	7	0.625	T（+）、RH（-）、V（+）、P（+）
	14	0.652	T（+）、RH（-）、P（+）
Wyoming	0	0.487	T（+）、RH（-）、V（-）、P（+）
	5	0.505	T（+）、RH（-）、V（-）、P（+）
	7	0.505	T（+）、RH（-）、V（-）、P（+）
	14	0.497	T（+）、RH（-）、V（-）、P（+）

Table S2. Results For Different Time Durations

Regions	Time duration	R2(PLSR)	Significant factors and correlations
UK	2020.1.31-2021.5.2	0.423	T（+）
	2020.1.31-2021.3.31	0.473	T（+）、RH（-）
	2020.1.31-2021.5.31	0.466	T（+）
Morocco	2020.3.2-2021.5.14	0.577	T（+）、RH（+）
	2020.3.2-2021.3.31	0.554	T（+）、P（-）

	2020.3.2-2021.5.31	0.558	T（+）、RH（+）
Nepal	2020.1.25-2021.2.28	0.716	T（+）、RH（+）、V（+）、P（-）
	2020.1.25-2021.1.31	0.683	T（+）、RH（+）、V（+）、P（-）
	2020.1.25-2021.3.31	0.743	T（+）、RH（+）、V（+）、P（-）
Uzbekistan	2020.3.15-2021.2.24	0.517	T（+）、RH（+）、P（-）
	2020.3.15-2021.1.31	0.492	T（+）、RH（+）、P（-）
	2020.3.15-2021.2.28	0.507	T（+）、RH（+）、P（-）
Wuhan	2020.1.14-2020.3.13	0.542	T（-）、RH（-）、P（-）
	2020.1.14-2020.3.31	0.425	T（-）、RH（-）、V（+）、P（-）
	2020.1.14-2020.4.30	0.150	T（-）、RH（-）、P（-）
Alabama	2020.3.13-2021.5.11	0.466	T（+）、P（+）
	2020.3.13-2021.3.31	0.491	T（+）、RH（-）
	2020.3.13-2021.5.31	0.417	T（+）
Arizona	2020.1.26-2020.10.1	0.254	T（-）、RH（-）、V（+）
	2020.1.26-2020.8.31	0.182	T（-）、RH（-）、P（-）
California	2020.1.25-2021.4.18	0.561	T（+）、RH（-）、V（-）、P（-）
	2020.1.25-2021.3.31	0.580	T（+）、RH（-）、V（-）、P（-）
	2020.1.25-2021.4.30	0.570	T（+）、RH（-）、V（-）、P（-）
Idaho	2020.3.13-2021.6.4	0.481	T（+）、RH（-）、P（+）
	2020.3.13-2021.4.30	0.586	T（+）、RH（-）、V（+）、P（+）
	2020.3.13-2021.5.31	0.514	T（+）、RH（-）、P（+）
Kentucky	2020.3.6-2021.4.29	0.363	T（+）、V（-）、P（+）
	2020.3.6-2021.3.31	0.404	T（+）、P（+）
	2020.3.6-2021.5.31	0.308	T（+）、RH（+）、P（+）
Massachusetts	2020.2.1-2021.7.29	0.286	T（-）、P（-）
	2020.2.1-2021.6.30	0.185	T（-）、P（-）
	2020.2.1-2021.8.31	0.430	T（-）、P（-）
Montana	2020.3.13-2021.4.26	0.338	T（+）、RH（+）、P（+）
	2020.3.13-2021.3.31	0.398	T（+）、P（+）
	2020.3.13-2021.5.31	0.282	T（+）、RH（+）、V（+）、P（+）
New Hampshire	2020.3.2-2021.7.14	0.173	T（+）、RH（+）
	2020.3.2-2021.5.31	0.264	T（+）、RH（+）
	2020.3.2-2021.6.30	0.208	T（+）、RH（+）
North Dakota	2020.3.11-2021.3.25	0.679	T（+）、RH（-）、V（-）、P（+）
	2020.3.11-2021.2.28	0.613	T（+）、RH（-）、P（+）
	2020.3.11-2021.4.30	0.614	T（+）、V（+）、P（+）
Oklahoma	2020.3.6-2021.5.29	0.300	T（+）、V（-）、P（+）
	2020.3.6-2021.3.31	0.322	T（+）、RH（+）、P（+）
	2020.3.6-2021.4.30	0.332	T（+）、RH（+）、P（+）
South Dakota	2020.3.10-2021.4.13	0.716	T（+）、V（-）、P（+）
	2020.3.10-2021.3.31	0.800	T（+）、P（+）
	2020.3.10-2021.4.30	0.660	T（+）、P（+）
Wisconsin	2020.2.5-2021.4.29	0.570	T（+）、RH（-）、P（+）

	2020.2.5-2021.3.31	0.625	T（+）、RH（-）、V（+）、P（+）
	2020.2.5-2021.5.31	0.501	T（+）、V（+）、P（+）
Wyoming	2020.3.11-2021.5.4	0.487	T（+）、RH（-）、V（-）、P（+）
	2020.3.11-2021.3.31	0.554	T（+）、RH（-）、V（-）、P（+）
	2020.3.11-2021.4.30	0.500	T（+）、RH（-）、V（-）、P（+）
